# Supplementary material for: Host range of naturally and artificially evolved symbiotic bacteria for a specific host insect
Source: mBio. 2024 Jul 31;15(9):e01342-24. doi: 10.1128/mbio.01342-24 (PMC11389372; doi:10.1128/mbio.01342-24)
Supplement: Supplemental material — Fig. S1-S9 and Table S1. [file mbio.01342-24-s0001.pdf]

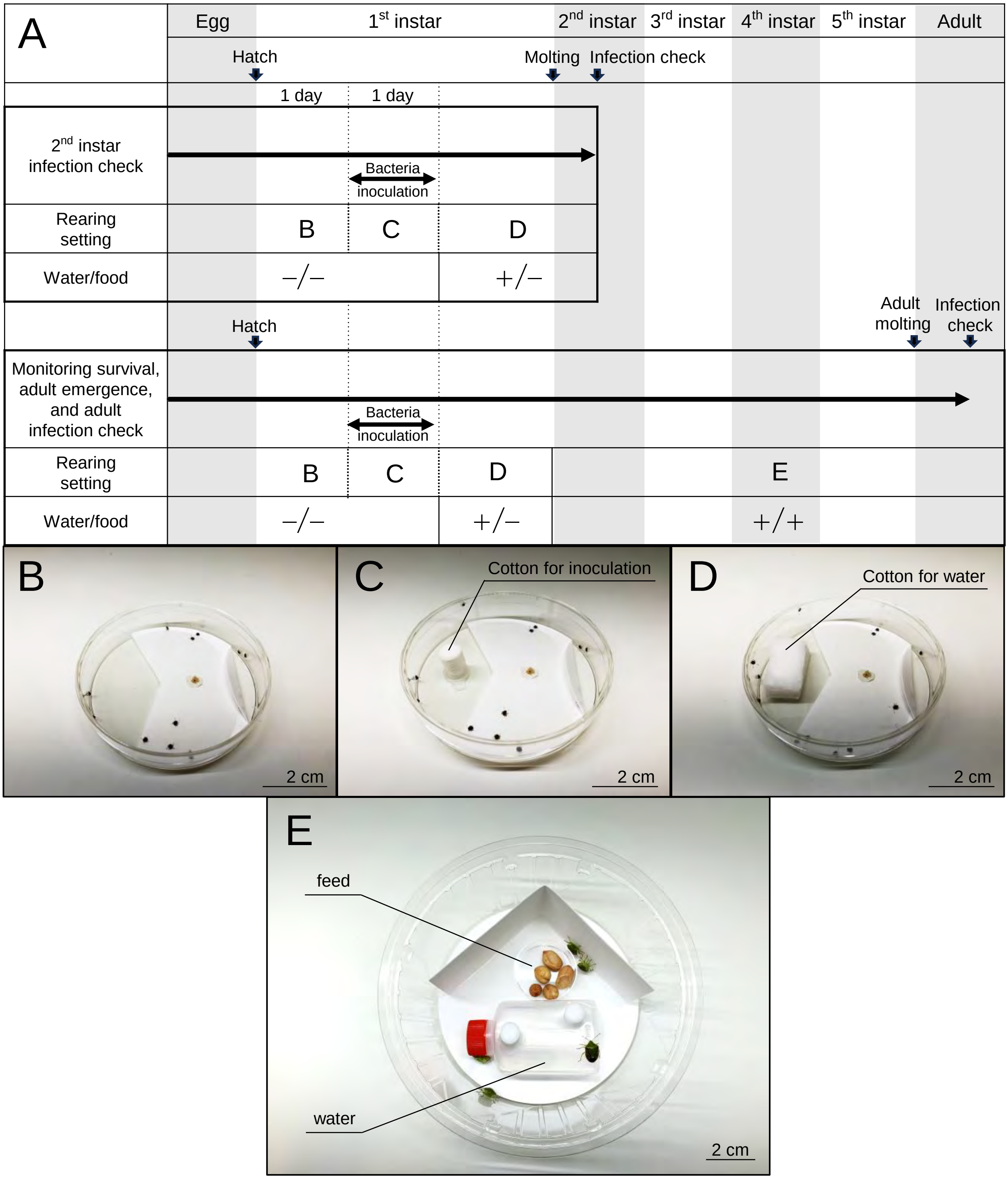

FIG S1 Insect rearing and bacterial inoculation procedures. (**A**) Procedures of insect rearing, bacterial inoculation, watering and feeding, etc. The upper half shows the procedures for 2nd instar infection check, whereas the bottom half shows the procedures for monitoring survival, adult emergence and adult infection check. (**B-D**) Rearing Petri dish settings for bacterial inoculation to symbiont-free newborn nymphs. (**B**) Before inoculation for 1 day without water. (**C**) During inoculation for 1 day with a cotton pad soaked with bacteria-suspended water. (**D**) After inoculation with a cotton pad soaked with sterile water. (**E**) Rearing container setting for monitoring survival, adult emergence and adult infection check with a water bottle and food seeds.

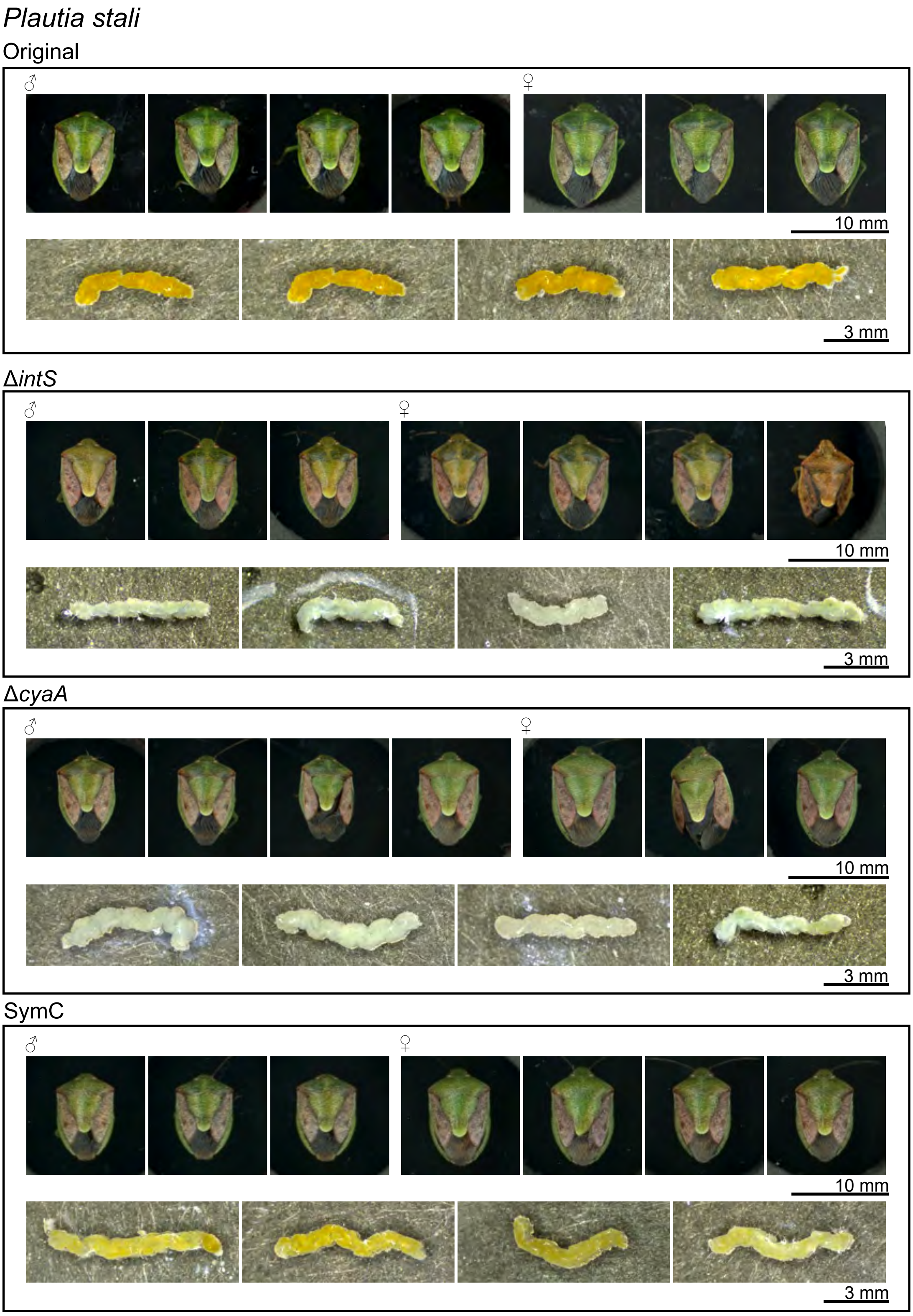

FIG S2 Adult insects of *P. stali* and their symbiotic organs obtained in this study. Also see Fig. 2.

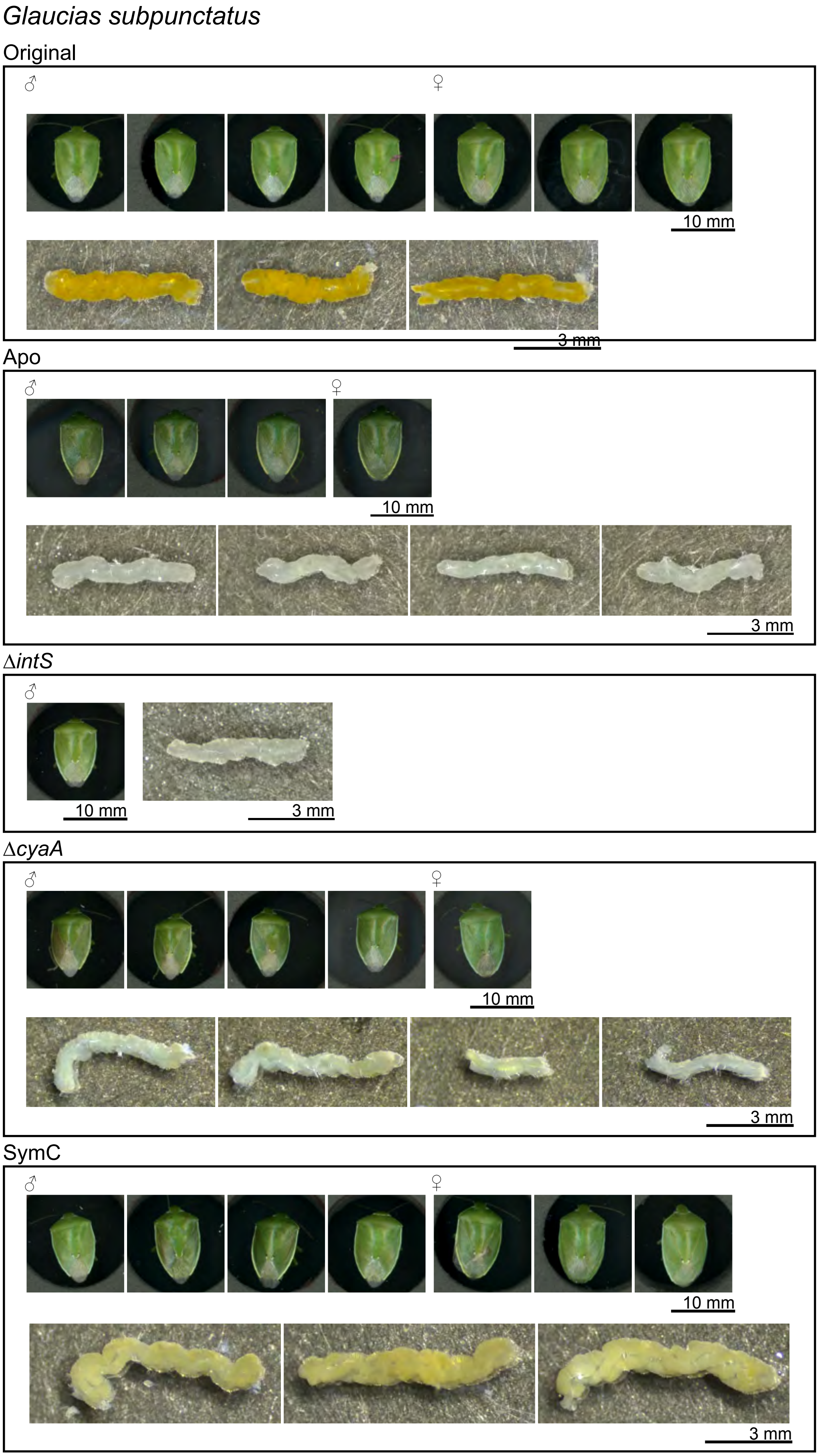

FIG S3 Adult insects of *G. subpunctatus* and their symbiotic organs obtained in this study. Also see Fig. 3.

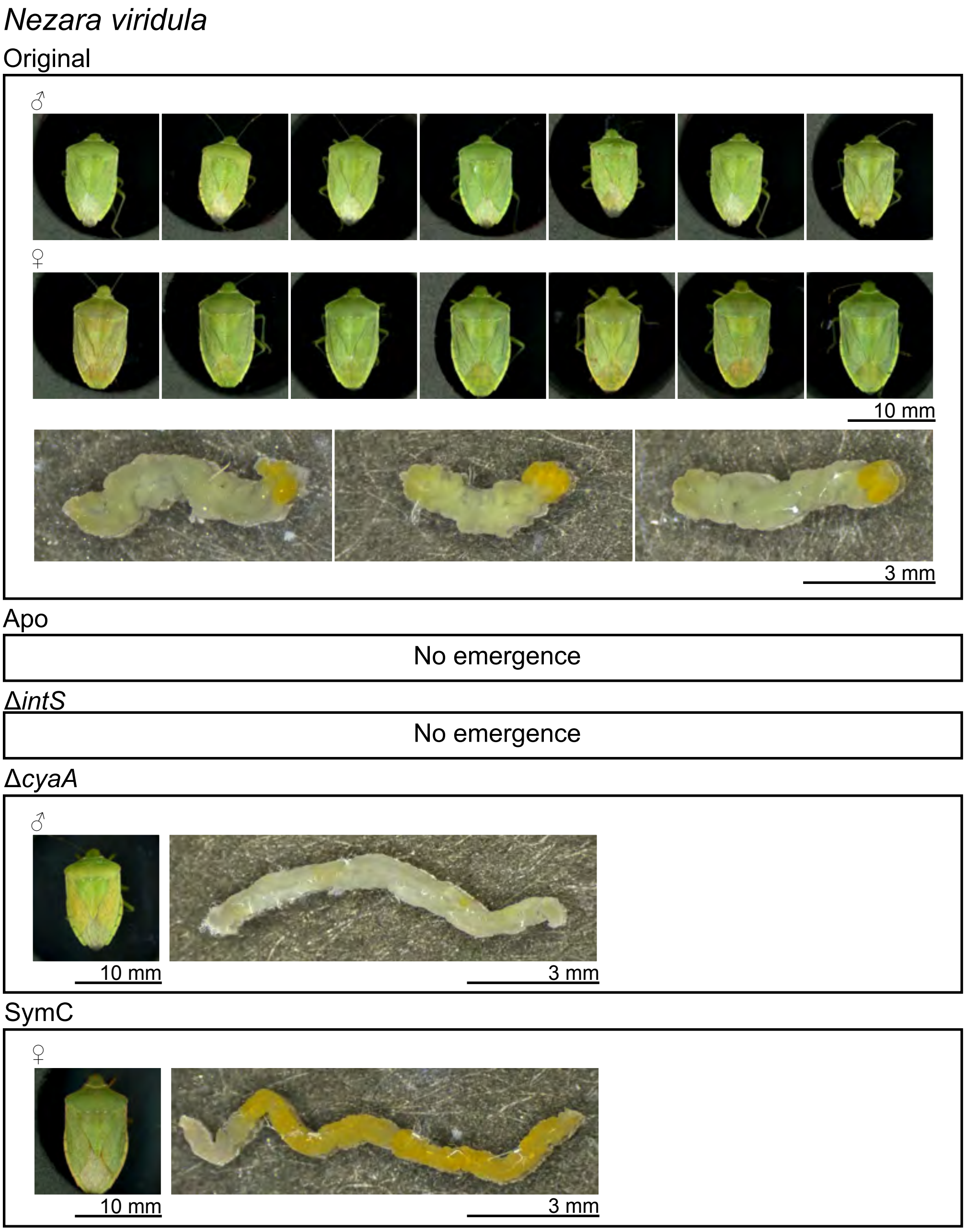

FIG S4 Adult insects of *N. viridula* and their symbiotic organs obtained in this study. Also see Fig. 4.

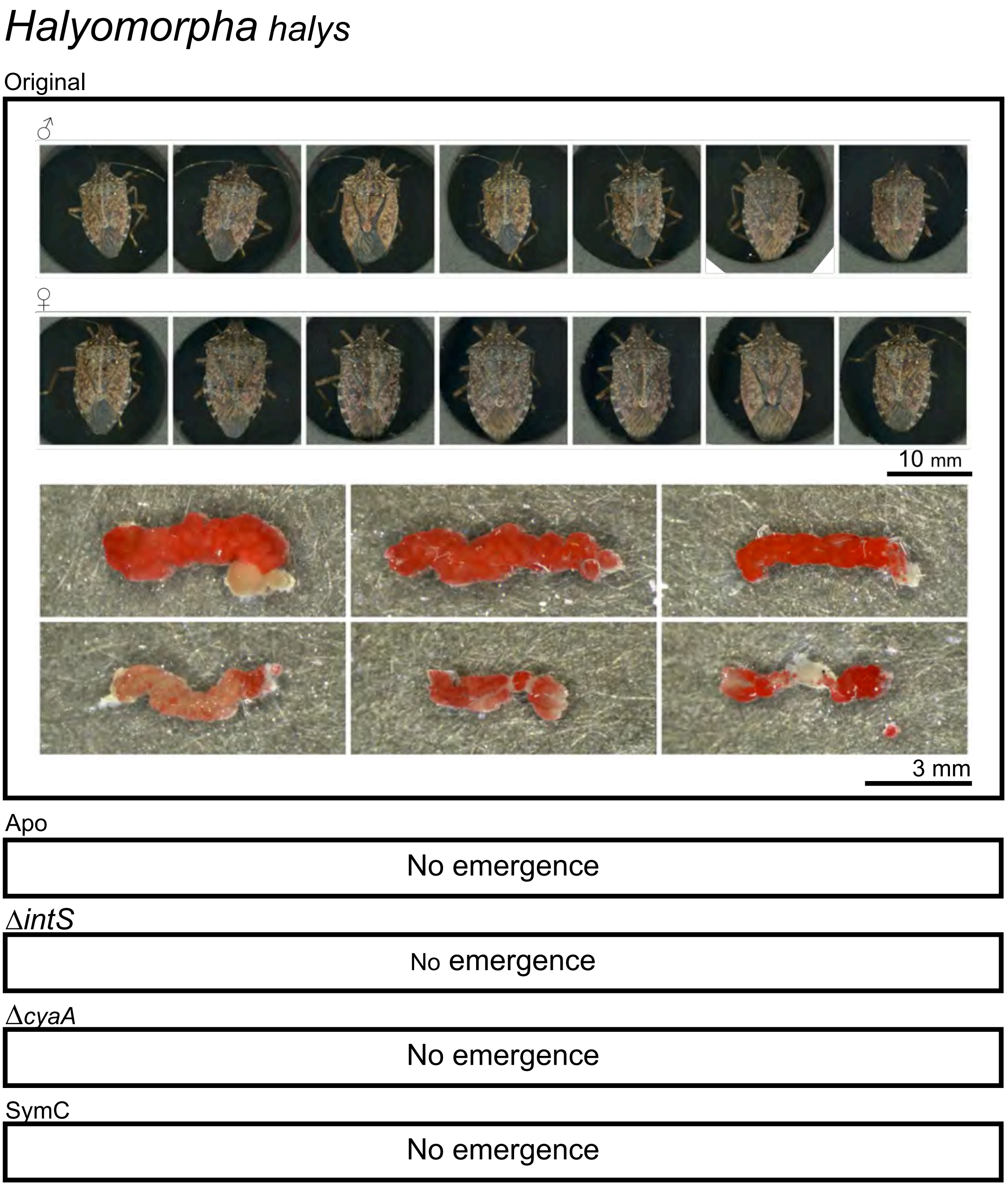

FIG S5 Adult insects of *H. halys* and their symbiotic organs obtained in this study. Also see Fig. 5.

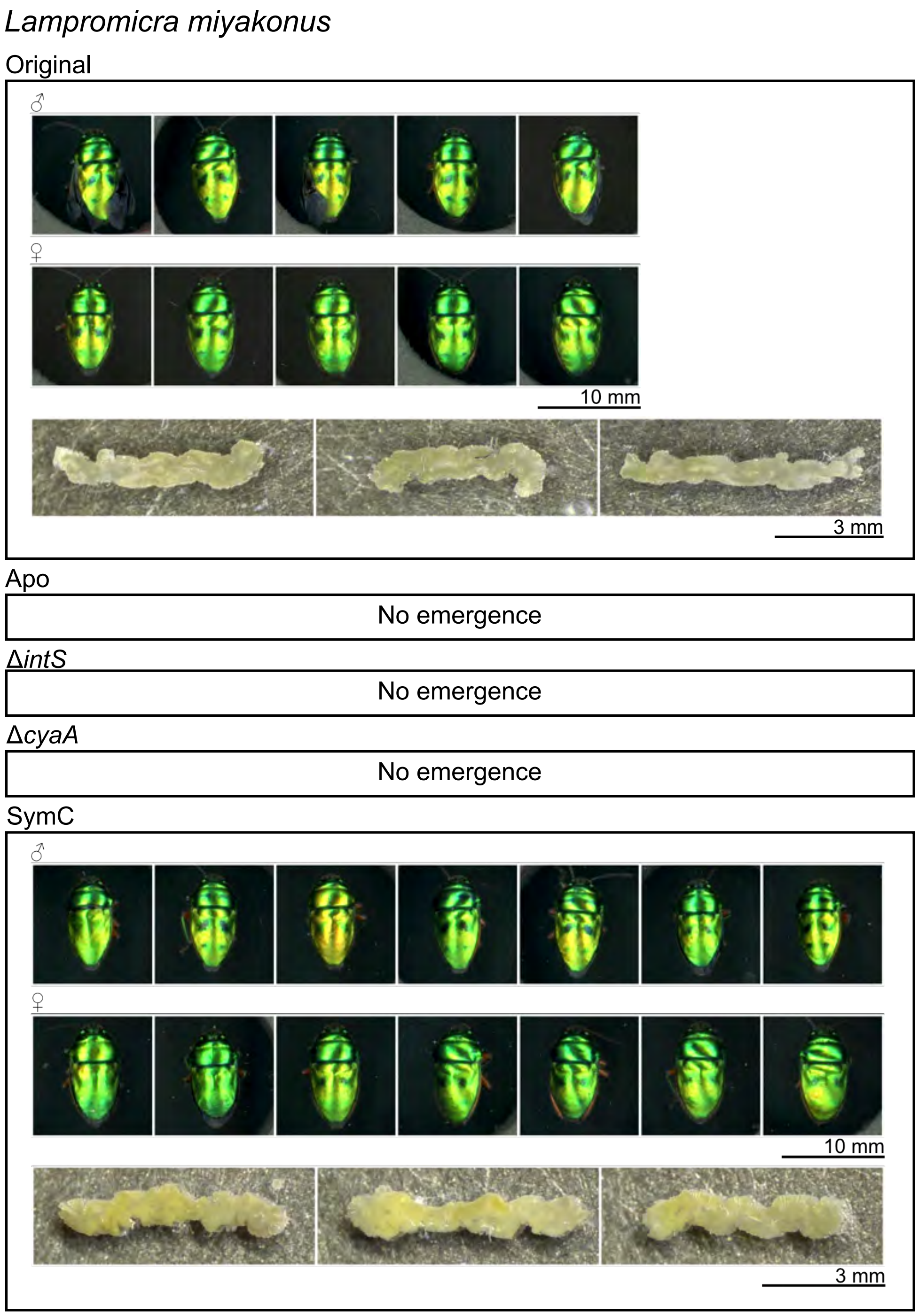

FIG S6 Adult insects of *L. miyakonus* and their symbiotic organs obtained in this study. Also see Fig. 6.

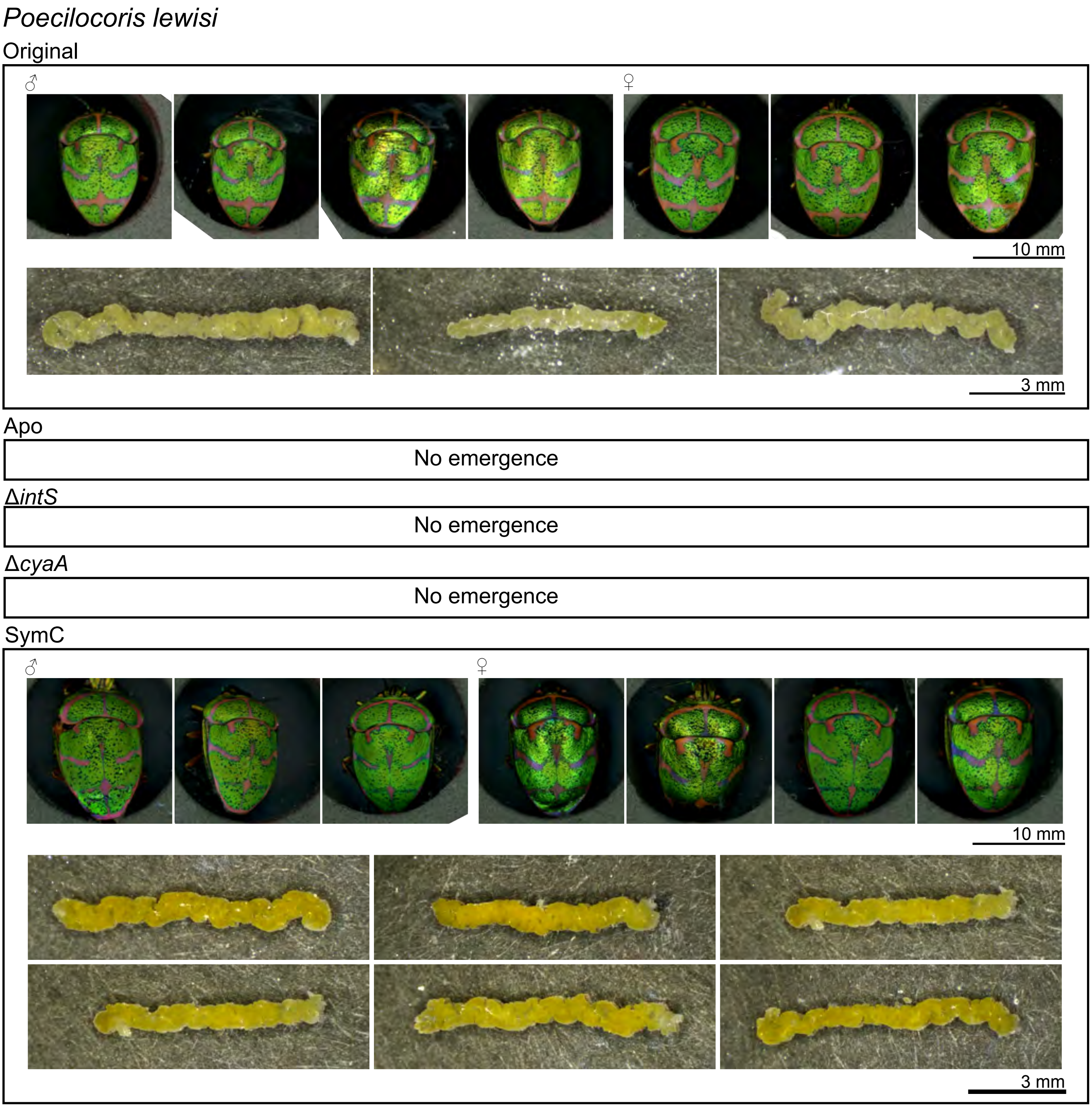

FIG S7 Adult insects of *P. lewisi* and their symbiotic organs obtained in this study. Also see Fig. 7.

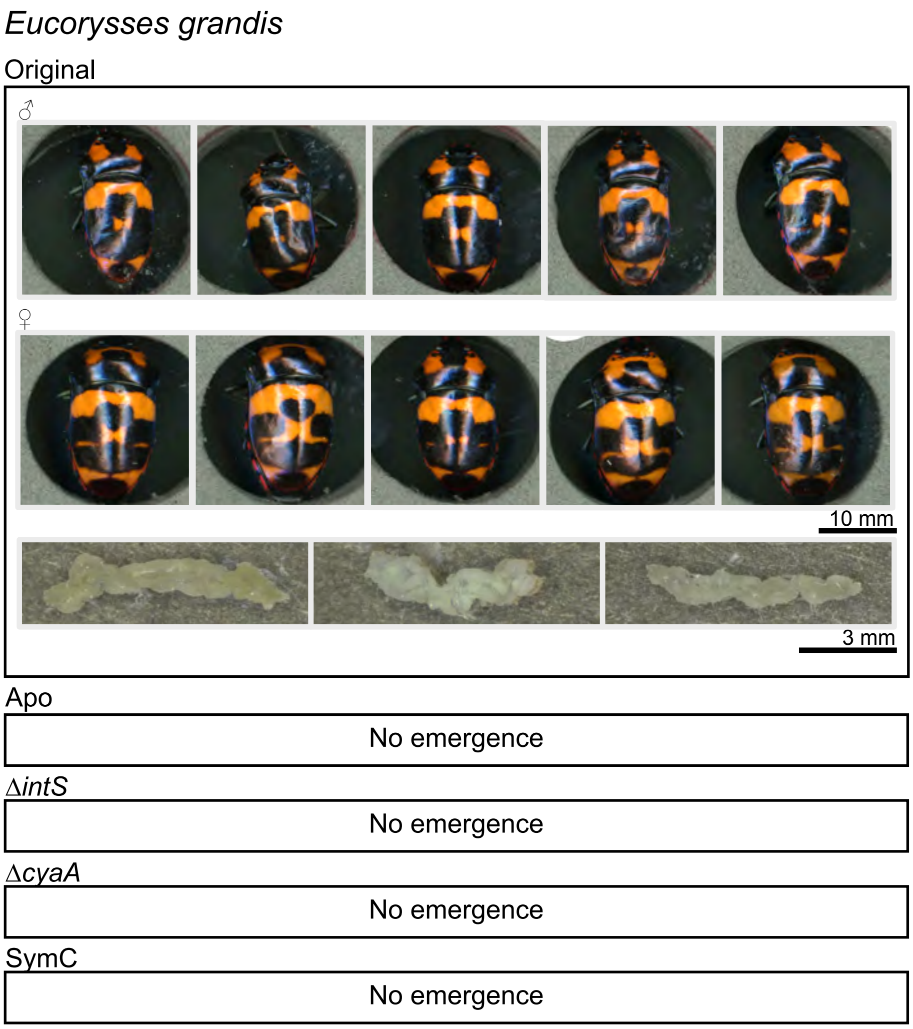

FIG S8 Adult insects of *E. grandis* and their symbiotic organs obtained in this study. Also see Fig. 8.

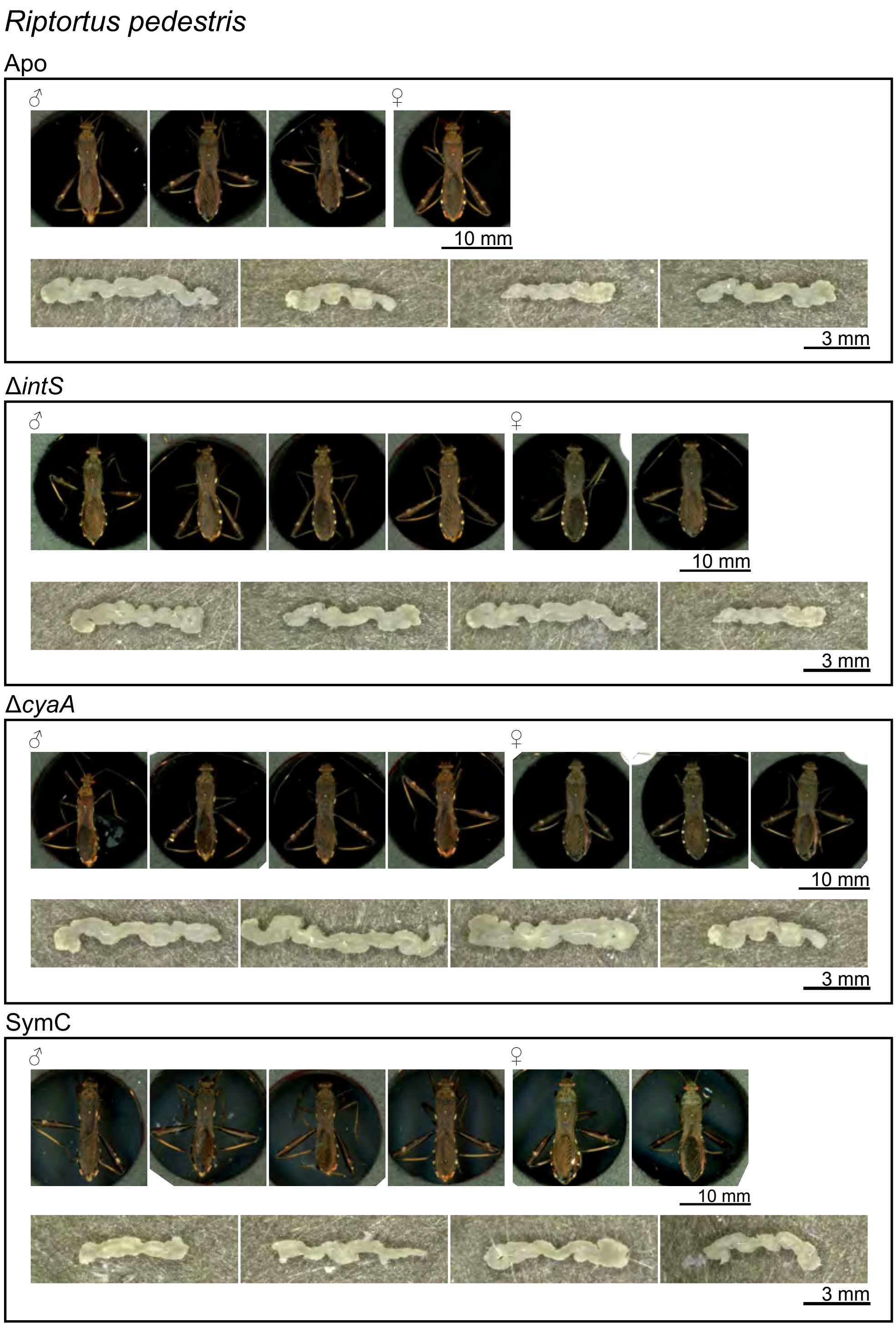

FIG S9 Adult insects of *R. pedestris* and their symbiotic organs obtained in this study. Also see Fig. 9.

Table S1. Laboratory strains of stinkbugs used in this study.

Insect	Symbiont	Symbiont reference	Collection locality	Collection year	Collector	Rearing condition	Food	Rearing reference	Rearing system	2nd instar infection check	Adult morphometry and infection check
**Family Pentatomidae**											
Plautia stali	Gammaproteobacteria Enterobacteriaceae *Pantoea* sp. A (Sym A)	Hosokawa et al. (2016a)	Tsukuba, Ibaraki, Japan	2012	Minoru Moriyama	16 h L, 8 h D, 25°C	Raw peanuts; Water with 0.05% vitamin C	Hosokawa et al. (2016a)	12-15 eggs per plastic cup	3 days after molting	41-64 days after hatch
Glaucias subpunctatus	Gammaproteobacteria Enterobacteriaceae Undescribed	Hosokawa et al. (2016b)	Tsukuba, Ibaraki, Japan	2018	Minoru Moriyama	16 h L, 8 h D, 25°C	Raw peanuts & raw almonds; Water with 0.05% vitamin C	This study	14-38 eggs per plastic cup	3 days after molting	40-67 days after hatch
Nezara viridula	Gammaproteobacteria Enterobacteriaceae Undescribed	Tada et al. (2011)	Kyoto, Kyoto, Japan	2019	Kaoru Ishida & Hideharu Numata	16 h L, 8 h D, 25°C	Raw peanuts & soybean seeds; Water with 0.05% vitamin C	Tada et al. (2011)	38-88 eggs per plastic cup	3 days after molting	45-67 days after hatch
Halyomorpha halys	Gammaproteobacteria Enterobacteriaceae Candidatus *Pantoea cerbekii*	Hosokawa et al. (2016b)	Tsukuba, Ibaraki, Japan	2017	Minoru Moriyama	16 h L, 8 h D, 25°C	Raw peanuts; Water with 0.05% vitamin C	This study	16-29 eggs per plastic cup	3 days after molting	56-101 days after hatch
**Family Scutelleridae**											
Lampromicra miyakonus	Gammaproteobacteria Enterobacteriaceae *Pantoea* sp. C (Sym C)†	Hosokawa et al. (2016a)	Ishigaki, Okinawa, Japan	2020	Minoru Moriyama	16 h L, 8 h D, 25°C	Raw almonds & raw cashew nuts; Water with 0.05% vitamin C	Hosokawa et al. (2016a)	8-15 eggs per plastic cup	3 days after molting	45-48 days after hatch
Poecilocoris lewisi	Gammaproteobacteria Enterobacteriaceae Undescribed	Hosokawa et al. (2019)	Ishigaki, Okinawa, Japan	2020	Minoru Moriyama	16 h L, 8 h D, 25°C	Raw almonds, raw cashew nuts Water with 0.05% vitamin C	This study	10-13 eggs per plastic cup	3 days after molting	50-76 days after hatch
Eucorysses grandis	Gammaproteobacteria Enterobacteriaceae Undescribed	Hosokawa et al. (2019)	Kamogawa, Chiba, Japan	2021	Bin Hirota	16 h L, 8 h D, 25°C	Raw almonds, raw cashew nuts Water with 0.05% vitamin C	This study	4-71 eggs per plastic cup	3 days after molting	60-76 days after hatch
**Family Alydidae**											
Riptortus pedestris	Betaproteobacteria Burkholderiaceae *Caballeronia insecticola*	Kikuchi et al. (2005)	Ishigaki, Okinawa, Japan	2021	Harumi Yamazaki	16 h L, 8 h D, 25°C	Soybean seeds Water with 0.05% vitamin C	Kikuchi et al. (2005)	3-20 eggs per plastic cup	3 days after molting	40-43 days after hatch

† Natural individuals of *L. miyakonus* harbor a cultivable gut bacterial symbiont closely related to Sym C of *P. stali*, whose experimental inoculation actually support normal growth and survival of *P. stali* (Hosokawa et al. 2016a).
